# *Gemella morbillorum* Endocarditis

**DOI:** 10.1155/2014/456471

**Published:** 2014-12-07

**Authors:** Serap Ural, Sureyya Gul Yurtsever, Bahar Ormen, Nesrin Turker, Figen Kaptan, Sibel El, Zehra Ilke Akyildiz, Nejat Ali Coskun

**Affiliations:** ^1^Department of Infectious Diseases and Clinical Microbiology, Katip Celebi University Ataturk Training and Research Hospital, Izmir, Turkey; ^2^Department of Clinical Microbiology, Katip Celebi University Ataturk Training and Research Hospital, Izmir, Turkey; ^3^Department of Cardiology, Katip Celebi University Ataturk Training and Research Hospital, Izmir, Turkey

## Abstract

Infective endocarditis caused by *Gemella morbillorum* is a rare disease. In this report 67-year-old male patient with *G. morbillorum* endocarditis was presented. The patient was hospitalized as he had a fever of unknown origin and in the two of the three sets of blood cultures taken at the first day of hospitalization *G. morbillorum* was identified. The transthoracic echogram revealed 14 × 10 mm vegetation on the aortic noncoronary cuspis. After 4 weeks of antibiotic therapy, the case was referred to the clinic of cardiovascular surgery for valve surgery.

## 1. Introduction

*Gemella morbillorum* is a bacterium that is present in the normal flora of human oropharynx, genitourinary system, and the gastrointestinal system [1]. Infective endocarditis caused by this microorganism is a very rare condition. Native valve endocarditis is reported more commonly relative to prosthetic valve endocarditis. Mitral and aortic valvular involvement are almost observed at the same ratios; sometimes, involvement of both may occur [2].

This paper investigated endocarditis caused by* G. morbillorum*, a very rare endocarditis factor based on the literature data. This is thought to be the first* G. morbillorum* endocarditis in Turkey.

## 2. Case Report

The 67-year-old male patient underwent gastroscopy and colonoscopy for investigational purposes under the preliminary diagnosis of anemia; the examinations revealed normal findings. After twenty days, the patient was referred to the infectious diseases outpatient clinic upon experiencing fever in addition to ongoing fatigue and deterioration of the overall status. The patient did not have preexisting pathologies such as cardiac diseases, steroid treatment, diabetes mellitus, and long-term antibiotic treatment or a history of intravenous drug abuse. The physical examination revealed that he was conscious with a body temperature of 38°C, blood pressure of 130/85 mmHg, a pulse of 85/min, and respiration of 23/min. Oral examination revealed the presence of total prosthesis. The cardiac and circulatory system examination detected 2/6° diastolic murmur in the aortic focus. The abdominal examination revealed dullness in the traube's space and palpable spleen at the rib curve. Results of the other system examination were normal. Laboratory test results were as follows: 8900/mm^3^, neutrophil: %78, hemoglobin: 10.1 gr/dL, erythrocyte sedimentation rate (ESH): 76 mm/hour, C-reactive protein (CRP): 114 mg/L (N: 0–5 mg/L), and procalcitonin: 0.14 ng/mL (N: 0–0.05 ng/mL). The results of the routine biochemical tests were normal. Flora bacteria grew in the throat culture while urinary culture showed no growth. Imaging tests revealed a normal lung graphy. The spleen size was detected to be increased (133 mm) at the abdominal ultrasonography (USG) with hypoechoic regions detected in the parenchyma at the sizes of 40 × 41 mm and 44 × 38 mm (infarction was considered primarily with abscess being considered secondarily). Computed tomography of the abdomen showed a spleen size larger than normal with the parenchymal hypodense lesions being considered as infraction areas or metastasis. Contrast-enhanced cerebral magnetic resonance imaging (MRI) showed a region that was consistent with subacute infraction in the left occipital lobe. Transthoracic echocardiography (ECHO) revealed an image that was consistent with vegetation of 14 mm × 10 mm size in the aortic valve (ımage). Electrocardiogram detected a normal sinus rhythm. Three hemocultures were obtained within the first 24 hours when the patient had fever. Incubation was performed at the Bactec 9240 (Becton Dickinson, USA). The samples obtained from the two hemoculture vials exhibiting positive signal were cultivated into the blood and chocolate media. In the blood agar, the bacteria that formed colonies resembling the small beta-hemolytic streptococcal colonies were detected to be oxidase-catalase negative Gram-positive cocci with further identification being performed by Phoenix (Becton Dickinson, USA). The susceptibility of the strain to penicillin (10 *μ*g), ampicillin (10 *μ*g), erythromycin (15 *μ*g), chloramphenicol (30 *μ*g), clindamycin (10 *μ*g), levofloxacin (5 *μ*g), linezolid (30 *μ*g), ceftriaxone (30 *μ*g), vancomycin (30 *μ*g), and teicoplanin (30 *μ*g) (Himedia, INDIA) was determined using the disc diffusion method based on the recommendations of the Clinical and Laboratory Standards Institute (using the streptococcus species as the standard) (CLSI) [3]; the bacterium identified as* G. morbillorum* was detected to be susceptible to all the antibiotics used.

**Figure 1:**
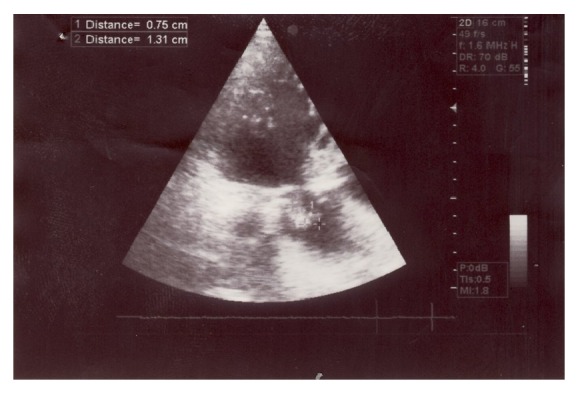
Vegetation on the aortic valve noncoronary leaflet (cuspis).

A decision to start medical treatment was made as a result of the cardiology and cardiovascular surgery clinic consultation. Under the diagnosis of infective endocarditis, since occurrence of abscess at the background of infraction in the spleen could not be excluded, treatment with ampicillin-sulbactam 12 grams/day IV and gentamicin 240 mg/day IV was started. After three weeks of treatment, the following values were detected: leukocyte: 5000/mm^3^, Hb: 8.99 gr/dL, ESH: 64 mm/hour, CRP: 55.2 mg/L, and procalcitonin: 0.80 ng/mL. Control ECHO showed vegetation of 7.5 mm × 13.1 mm size in the aortic noncoronary cuspis (Figure 1). The control abdominal USG showed a spleen size that was at the upper limit of the normal with two hypoechoic lesions in the parenchyma with a size of 61 × 39 mm and 39 × 37 mm, respectively. A repeated cardiology and cardiovascular surgery consultation was requested for the patient, who showed a partial reduction in the body temperature that could not be completely kept under control with no reduction in vegetation, upon the decision to continue with the medical treatment. Cardiovascular surgery patients despite the recommendations and guidelines of all our insistence operation did not take. Our view was in favor of early operation. The previous medication was discontinued and replaced by a broader-spectrum meropenem 6 grams/day IV and vancomycin 2 × 1 gram/day IV. The patient, receiving this treatment for a week, developed left hemiplegia after 4 weeks. The cerebral diffusion MRI performed on the same day detected an early-stage infraction in the right frontotemporal region. The patient for whom a decision to perform valvular replacement was made was referred to an external site for operation by his own will.

## 3. Discussion

While* G. morbillorum *described for the first time by Tunnicliff in 1917 and classified as* Streptococcus morbillorum *is present in the genitourinary system and gastrointestinal system flora, it may rarely lead to infections including infective endocarditis, meningitis, cerebral abscess, pneumonia, and peritonitis. Twenty-four cases of endocarditis were reported in the literature up to 2010 [2]. Predisposing factors for endocarditis include dental hygiene, dental procedures, colon malignities, inflammatory intestinal disorders, gastrointestinal diagnostic procedures such as colonoscopy, preexisting cardiac pathologies (valvular lesions, hypertrophic cardiomyopathy, and cardiac myxoma), steroid treatment, and diabetes mellitus [2, 4, 5]. While investigating anemia in our patient, gastroscopy and colonoscopy were detected to be predisposing factors.

A majority of the* G. morbillorum* isolates isolated from various clinical samples are reported to be susceptible to penicillin G and ampicillin. However, in the subsequent years, several penicillin- and macrolid-resistant strains were detected [4]. In most of the cases, bacteriologic cure was achieved with penicillin G + aminoglycoside combination. Vancomycin or erythromycin + rifampicin combination is effective in the presence of in vitro penicillin resistance or in beta-lactam allergic cases [6]. IV vancomycin was used for 6 weeks in two beta-lactam and aminoglycoside-resistant cases; while one achieved a complete cure, the other underwent valvular replacement upon experiencing septic emboli [1]. Linezolid may be used in cases of penicillin, aminoglycoside, and vancomycin resistance [5]. In our case,* G. morbillorum* was susceptible to all antibiotics in the antibiogram; however since the spleen abscess could not be excluded, treatment with ampicillin-sulbactam + gentamicin was preferred. The patient was shifted to meropenem + vancomycin treatment upon failure to achieve inadequate response to this treatment. However operation was decided to be performed since acute cerebral emboli developed.

In a penicillin-resistant patient, who developed agranulocytosis with piperacillin and acute renal failure with cefoperazone sodium, 4-week cefotiam dihydrochloride treatment was administered; however cure was achieved by performing aortic valvular replacement upon occurrence of cardiac failure [6]. ECHO performed upon detecting persistent fever in a Japanese patient, who has received dental treatment 6 months ago, revealed vegetation on the aortic and mitral valve and penicillin G 18 million U/day and diuretic and cardiotonic agent treatment was initiated. The patient with no response to medical treatment, who developed penicillin-associated granulocytopenia on the 35th day and underwent appendicitis operation, was discharged on the 101st day following mitral and aortic valvular replacement. This was reported to be the first case of* G. morbillorum* endocarditis in Japan [7].

Urgent surgical treatment is recommended in cases of progressive cardiac failure, large vegetations that could be an embolic source, reaction to antibiotics, and lack of response to antibiotic treatment [1, 5, 6]. In a 67-year-old male patient undergoing hemodialysis due to chronic renal failure, medical treatment with intravenous ampicillin and gentamicin was initiated upon occurrence of* G. morbillorum*-associated aortic and mitral valve endocarditis; decision to continue this treatment for a couple of weeks before surgery was made; however the patient died due to acute myocardial infraction [5]. In our patient for whom medical treatment was planned by the cardiovascular surgery clinic, no reduction in vegetation was detected despite the 4-week treatment, and surgical treatment was indicated upon occurrence of emboli.

A case of* G. morbillorum* endocarditis in a patient with human immunodeficiency virus infection and intravenous substance addiction, who had received mitral valvular replacement 9 months ago, was successfully treated with ceftriaxone (4 weeks) and gentamicin (2 weeks) [8]. Similarly, in a patient, who had previously received valvular replacement with the diagnosis of congenital bicuspid aortic valve, penicillin G and rifampicin were administered for 6 weeks and gentamicin was administered for 2 weeks with the diagnosis of endocarditis established upon* G. morbillorum* growth and complete recovery was achieved [4]. In a case report of prosthetic valvular endocarditis by Obadah Al Chekakie [9],* G. morbillorum* was reported to potentially lead to progressive and life-threatening cardiac failure. A 87-year-old male patient was treated under the diagnosis of* G. morbillorum*-associated native valvular endocarditis with no predisposing factor detected [10].

In conclusion, physicians should consider the fact that rare pathogens such as* G. morbillorum *could lead to endocarditis, particularly in the presence of predisposing factors. The potential requirement for urgent surgical treatment despite a good response to medical treatment should also be kept in mind.
